# Weight-Dependent Disparities in Adolescent Girls: The Impact of a Brief Pilot Intervention on Exercise and Healthy Eater Identity

**DOI:** 10.3390/ijerph15071411

**Published:** 2018-07-04

**Authors:** Eydie N. Kramer, Christine A. Chard, Kellie Walters, Daheia J. Barr-Anderson

**Affiliations:** 1 School of Kinesiology, University of Minnesota, Minneapolis, MN 55455, USA; barra027@umn.edu; 2 Department of Health and Exercise Science, Colorado School of Public Health, Colorado State University, Fort Collins, CO 80523, USA; Chrissy.chard@colostate.edu; 3 Department of Kinesiology, California State University, Long Beach, CA 90840, USA; Kellie.walters@csulb.edu

**Keywords:** obesity, adolescents, health behavior, physical activity, diet, identity, health disparities

## Abstract

Adolescent girls report low participation in healthy behaviors (e.g., nutritious eating and exercise), and are disproportionately affected by obesity. Short-term interventions, such as behavioral summer camps, may positively influence psychological underpinnings of healthy behavior, particularly exercise identity (EI) and healthy eater identity (HEI). The present study investigates disparities and changes in identity and subsequent health behavior in two cohorts of adolescent girls following a brief, multicomponent intervention. A sample of normal-weight adolescent girls from a health promotion camp and an elevated body mass index (BMI) sample from an obesity treatment camp participated in the study. Both camps ran one-week in duration and delivered comparable intervention components. All families were given access to the same eight-week eHealth program post-camp. Significant EI and HEI role-identity disparities between the health promotion and obesity treatment cohorts were apparent at baseline. Following the one-week camp intervention, EI and HEI scores increased in both groups. At follow-up, the treatment group had increased EI and HEI role-identities in such that the groups no longer significantly differed. Positive changes in health behaviors were experienced in each group. This pilot study demonstrates that EI and HEI differ between normal-weight and obese adolescent girls and weight-dependent identity disparities may be mitigated following brief, multicomponent interventions.

## 1. Introduction

Despite a recent plateau in the upward trend, childhood and adolescent obesity rates remain high in the United States (U.S). Recent analyses reveal age-specific prevalences of obesity (defined as body mass index (BMI) at or above the sex-specific 95th percentile on U.S. Centers for Disease Control and Prevention (CDC) growth charts) as follows: 8.9% among children ages 2–5, 17.5% among children ages 6–11, and 20.5% among adolescents ages 12–19 [1]. A variety of health consequences disproportionately affect obese youth, and therefore the high prevalence of this condition represents a substantial public health concern. The risk of developing cardiovascular disease, asthma, and non-smoking related cancers in adulthood may be increased if an individual has pediatric obesity [2]. Psychological health may also be negatively impacted, as children who are categorized as obese at young ages (4–5) possess a heightened risk of developing emotional health conditions in later childhood years [3]. One strategy to ameliorate the potential health disparities youth with elevated weight status face is through the delivery of interventions which target sex-specific gaps in health behavior (i.e., nutritious eating and physical activity behavior disparities), as well as the psychological variables influencing such behavior.

A large percentage of adolescent girls in the United States do not meet the minimum physical activity guidelines or engage in recommended healthy dietary behaviors and are disproportionately overweight or obese as compared to their male counterparts [4]. Taken together, the evidence which supports a connection between obesity in adolescence and numerous physical, psychological, and emotional health risks [5,6] and apparent sex-specific differences in health behavior demonstrate a need for unique behavior change interventions for adolescent girls. Notably, behavioral interventions often strive to engage individuals in lifestyle strategies to: (1) combat potential health deficits linked to inactivity and poor dietary habits, and (2) propagate long-term habits which enhance health across the lifespan. Of particular interest is the development of psychological variables, such as health identity, in the adolescent years which underpin healthy lifestyle choices. Strongly held health identities are conducive to long-term health behavior maintenance and may be conceptualized as an individual’s exercise identity (EI) or healthy eater identity (HEI). Preliminary evidence indicates that EI is associated with enhanced exercise behavior in adult populations [7,8], and HEI is linked to nutritious dietary choices (i.e., increased fruit and vegetable intake and less red meat consumption) in both adult and adolescent populations [9,10,11,12]. The apparent physical activity and healthy dietary behavior disparities among normal-weight and obese youth may indicate that obese youth lack identities that support self-regulation of, and adherence to, consistent exercise participation and nutritious eating patterns. Indeed, perception of oneself as ‘fat’ may result in internalization of weight stigma, equivocation of a ‘fat’ identity to a ‘physically unfit’ identity, and subsequent avoidance of healthy behavior [13,14].

The theoretical foundations of EI and HEI lie in identity theory. Individuals hold salient knowledge and beliefs about their unique social roles, within the context of encompassing social categories or groups [15]. It is thought that a strong role-identity results in predictable behaviors, as individuals strive to behave in ways which are congruent with portrayals of their chosen identity standard [15,16]. Individuals with a particularly strong role-identity may seek out social groups and environments which support behaviors linked to the meaning of their perceived role. These role meanings (i.e., what an individual believes it means to be an ‘exerciser’ or a ‘healthy eater’), consistently guide and influence behavior, and may even impact future perceptions of self [15]. For example, an adolescent girl who does not regularly engage in physical activity may not identify as an ‘exerciser’ and may not seek out situations in which to participate in exercise in her daily routine (i.e., avoid physically active environments or lack interaction with physically active individuals). Therefore, it is unlikely that the role-identity of ‘exerciser’ will increase. Inversely, an adolescent girl who embraces healthy behavior change and adopts a lifestyle conducive to regular physical activity may begin to see exercise as an integral part of her social role and consequently strengthen her identity as an ‘exerciser’. This increase in EI salience may further enhance motivation to participate in physical activity, even when the individual is faced with situational barriers to health behavior engagement. Thus, the relationship between identity and behavior appears interconnected, and reciprocal [15]. It is clear that an individual’s identity, including that of an ‘exerciser’ or ‘healthy eater’, may be developed or strengthened in an evolving sequence across the lifetime. However, the question remains, can EI or HEI be meaningfully impacted in a phase of life which presents high-risk for inactivity, poor dietary patterns, overweight, and obesity: the adolescent years? The stages of identity development in adolescence have been examined extensively in the literature. Waterman’s hypothesis of identity development states: “Movement from adolescence to adulthood involves a preponderance of changes in identity status which can be characterized as progressive developmental shifts” [17]. Indeed, research indicates that identities are most actively formed in adolescence and early adulthood, and are relatively stable once developed [17,18].

Current trends indicate that the summer months present a high-risk for poor health behavior and weight-gain as compared to the academic year for overweight and obese youth [19], and the U.S. Preventative Services Task Force recommends engagement in behavioral programs as an early treatment option for obesity [20]. Therefore, immersive behavior change summer camps may be an ideal intervention format to enhance health identities conducive to exercise and healthy dietary choices. Immersion programs which include an exercise component appear to produce favorable results for long-term weight control, especially when strong commitment levels to healthy behaviors, cognitions, and attitudes have been developed [21]. Another innovative strategy to increase health identity in adolescence is to deliver health programming through an eHealth intervention. Technology-based interventions may be especially appropriate, as a steady increase in computer accessibility and use of handheld devices (i.e., smartphones) has been seen in the past few decades [22]. Specifically, 95% of adolescents report using the internet [23], and over three-quarters of households in the United States report internet use on personal computers, smartphones, or tablets [24]. Numerous studies have examined the effectiveness of eHealth programs aimed at preventing obesity and increasing healthy behaviors in child and adolescent populations. The results have been largely positive and offer a potential health intervention strategy [25,26,27,28,29]. More research is needed to evaluate eHealth intervention effects in the long-term.

Several trials have utilized unique modes of technology as intervention components when testing the impact health identity may have upon targeted health behaviors. In two recent studies investigating the effect of psychological variables on reducing red meat consumption, the influence of healthy-eating and meat-eating identities on food intake intentions and behaviors were examined. Utilizing a cross-sectional design, researchers collected a one-week online food diary and survey data measuring past behavioral control, intentions, and identity from a convenience sample of university students. Results indicated that a lower meat-eating identity was a predictor of intentions to reduce red meat consumption, while a higher healthy-eating identity was not [12]. However, these results should be interpreted with caution when considering the potential implications of health identity on behavior change, due to the cross-sectional design of the trial. Notably, the researchers conducted a subsequent randomized control trial in which a convenience sample of university students also completed food diaries and submitted data from online questionnaires and were then randomly assigned to two conditions (intervention versus control). The intervention cohort received reminders delivered via text message to monitor red meat intake, and the control group did not; the text message intervention was administered on seven consecutive days. Analyses demonstrated that healthy-eating identity mediated intentions to reduce red meat consumption [12]. Findings from aforementioned study which indicated that a HEI may meaningfully influence eating behavior are in line with previous research [9]. Limitations of previous investigations in the field of health identity development for behavior change include: (1) no immersive, face-to-face interventions have been utilized in combination with a digital intervention; (2) trials were not conducted in adolescence, the adolescent years are a time-frame in which identity is most saliently developed; and (3) potential differences in health identity based upon weight status have not been previously examined.

No published studies have examined the effects of a multicomponent behavior change summer camp plus eHealth intervention on health identity in two separate cohorts of adolescent girls: normal-weight versus BMI classified as overweight or obese adolescent. The present pilot study investigated the effects of a brief, multicomponent intervention on adolescent girls’ EI, HEI, and health behavior immediately following participation in an immersive one-week health camp and at follow-up. We hypothesized that (1) differences in identity would exist between a normal-weight cohort and an obese cohort at baseline, and (2) weight-dependent identity disparities would be mitigated following the intervention.

## 2. Materials and Methods

A quasi-experimental, pretest–posttest design was utilized for the present pilot study. Approval for the study was obtained from University of Minnesota and Colorado State University institutional review boards, and all participants and caregivers provided assent and consent, respectively. Two behavioral summer health camps located in Colorado (CO) and Wisconsin (WI) were selected as recruitment sites, based upon the target population of each camp. Overweight or obese BMI classification was not required for camp eligibility in the CO camp (hereafter referred to as ‘health promotion camp’) vs. overweight or obese BMI classification was required for camp eligibility in the WI camp (hereafter referred to as ‘obesity treatment camp’). Twenty-one adolescent girls from the health promotion camp and 20 participants were from the obesity treatment camp were recruited.

Both camps delivered comparable physical activity and nutritional education intervention components and ran one-week in duration during the summer of 2017. Primary post-camp outcomes were EI and EI role-identity (ERI) and HEI and HEI role-identity (HERI). Following camp, all participants were given access to the same eight-week eHealth program. The eHealth program focused upon promoting exercise and healthy eating behaviors in the home environment. The ultimate goal of the eHealth program was to maintain EI and HEI outcomes enhanced by the one-week behavioral health camps. Participation in the eight-week eHealth program following camp was encouraged, but not mandatory for study participation. Data were collected at baseline, post-camp, and immediately following the eight-week eHealth program.

### 2.1. Recruitment and Camp Programming Description

**Figure 1:**
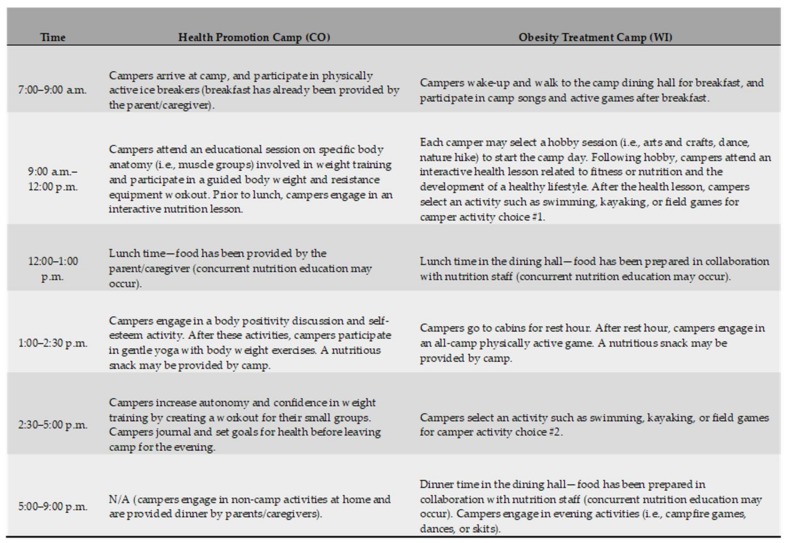
Overview of camp programming provided at the health promotion cohort (Colorado, CO) and the obesity treatment cohort (Wisconsin, WI). Total time spent in physical activity: 2–3 h (CO); 2–4 h (WI). N/A: not applicable.

Eligible participants were adolescent girls who self-selected attendance at either the health promotion or obesity treatment camp (based upon location, and services advertised by each specific camp) in summer 2017. Camp directors and research staff collaborated to deliver recruitment emails to participating camp families prior to each camp session, and research staff further distributed recruitment materials at camp informational sessions. The health promotion camp programming was adapted from a research-based after-school program (Smart Fit Girls) whose mission is to foster self-esteem and body image in adolescent girls through resistance training instruction, nutritional education, and positive psychology lessons. Programming delivered at the health promotion camp has been evaluated 11 times at various research sites across the United States, and results from evaluation data indicate that participants report significant increases in self-esteem, and improvements in body image and physical activity enjoyment as compared to comparison controls [30]. Weight management was not a focus of programming for the health promotion camp. The obesity treatment camp programming was created by weight-management medical staff from a local Wisconsin health-care system, and a majority of participants were referred to camp by their family practitioner. The mission of the treatment camp was to provide an active and fun interventional experience for campers with overweight or obesity, with the ultimate goal of creating healthy lifestyle behaviors related to physical activity, nutrition, and positive psychology. Weight management was a focus of programming for the obesity treatment camp. Staff at both camps consisted of exercise science, nutrition, or medical/behavioral psychology professionals. All camp staff passed background checks and engaged in comprehensive training prior to camp. A comparison between a typical day at camp demonstrates several differences and similarities in programming, based upon the unique needs and mission of each camp (Figure 1).

### 2.2. Multicomponent Intervention Description and Data Collection

Study participant assent and adult caregiver consent was required for inclusion into the study. Exclusion criteria included inability to access the optional eight-week eHealth program post-camp, and self-exemption from the study at any time point between baseline and follow-up. Baseline and immediate post-camp data were collected by trained research staff. Following participation in the one-week camp, families from both the health promotion and obesity treatment camps were given access to the same eHealth program. Camp families received reminder emails following camp to begin the eHealth program on the same date at the end of August 2017, to ensure that follow-up measures would be collected during the same time frame within the academic school year across the two cohorts (at approximately three-months follow-up). The eHealth program consisted of an introductory period which lasted 1 week in duration, and eight weekly eHealth lessons. The weekly curriculum correlated to camp programming and focused on physical activity foundations (including guidelines for weekly exercise participation for both adults and adolescents), nutritional education (including dietary guidelines), and positive psychology lessons. Additionally, each of the eight weekly eHealth lessons also introduced a ‘health challenge’ for the adolescent girls and their families to complete and report on before the start of the next lesson. Participating families received weekly encouragement emails, prompting participation in the eHealth program. Online traffic of the eHealth program was digitally measured on a weekly basis, and adult caregivers were given the option to respond to weekly evaluation surveys regarding the current health lesson. Follow-up data were collected by trained research staff at the culmination of the eHealth program in October 2017.

### 2.3. Study Measures

Study measures were collected at baseline (prior to beginning of camp programming), immediately following participation in the one-week behavioral health camp, and at three-month follow-up (immediately following the culmination of the eight-week eHealth program). The primary outcomes were health identity (EI, HEI) and role-identity (ERI, HERI), and the secondary outcomes were physical activity levels and dietary behavior (i.e., fruit, vegetable, and sugar-sweetened beverage intake). As weight was not an outcome of this study and the participants in the health promotion cohort attended a camp focused upon improving body image and self-esteem, height, and weight were not measured by research staff at camp. Rather, research staff calculated BMI with self-report height and weight data from both camps. In the obesity treatment camp, medical staff collected anthropometric data confirming camp attendance weight eligibility (BMI classified as overweight or obese) at baseline. All medical staff were trained in standard anthropometrical assessment methodology, according to the facilitating healthcare system’s requirements.

Exercise identity (EI) outcomes were assessed utilizing the validated exercise identity scale (EIS). The EIS indicates importance placed upon exercise as an integral part of an individual’s overall self-identity and has been validated by previous studies [31]. Each participant’s EI score was calculated by summing nine items measured on a seven-point Likert-type scale (with a range of 9–63) in response to statements such as, “I consider myself an exerciser; I have numerous goals related to exercise; I would feel a real loss if I was forced to give up exercising” (‘strongly disagree’ to ‘strongly agree’). The EIS may be divided into two sub-components: Exercise Beliefs (EB: EIS questions 3–5, 7–9) and Exercise role-identity (ERI: EIS questions 1,2,6). While both ERI and EB have been associated with exercise behavior and perceptions of physical activity, research indicates that role-identity may be a stronger predictor of frequent exercise participation than the beliefs measure [32]. Healthy eater identity (HEI) outcomes were measured utilizing healthy eater identity scale (HEIS). The HEIS was developed by Strachan & Brawley [11,33] and measures an individual’s self-perception of healthy eating as a vital part of their self-identity. The HEI is scored exactly the same as the EIS, and therefore is inclusive of an overall score ranging from 9–63, and two sub-component scores (healthy eater beliefs, HEB; healthy eater role-identity, HERI). Similar to the EIS, the HEIS consists of items such as, “I consider myself to be a healthy eater; Other people see me as someone who eats a healthy diet often; Eating a healthy diet is very important to my perception of myself” (seven-point Likert scale ranging from ‘strongly disagree’ to ‘agree’). The Cronbach alpha coefficients of EI, ERI, HEI, and HERI for the groups ranged from α = 0.90–0.94, and thus demonstrated acceptable internal consistency. Physical activity intensity levels (strenuous, moderate, or mild) were estimated using self-report data from the modified Godin–Shephard Leisure-Time Physical Activity Questionnaire (GSLTPAQ). The GSLTPAQ is a four-item survey which seeks to estimate the time an individual participates in physical activity each week, stratified by various levels of physical activity intensity. The GSLTPAQ been validated in adult populations and been utilized in numerous studies targeting exercise behavior in youth [34]. In line with methodology utilized in previous studies [34], data from the modified GSLTPAQ was transformed from time ranges spent in physical activity to quantifiable metrics (for example, “Participated in ½–2 h of strenuous activity per week” was transformed to “1.33 h per week”). Questions evaluating weekly participation in aerobic and resistance training activities and intake of fruits, vegetables, and sugar-sweetened beverages were drawn from the 2013 Youth Risk Behavior Survey (YRBS) National Questionnaire [35].

### 2.4. Data Analyses

Descriptive statistics for the present study are presented as means and standard deviations or frequencies and proportions (when appropriate). Statistical analyses were performed utilizing statistical analysis software (SAS) 9.4 TS Level 1M3 (SAS Institute, Cary, NC, USA), and significance level was set at *p* < 0.05. Between-group differences (health promotion vs. obesity treatment cohort) in baseline demographic and health identity characteristics were assessed using two-sample *t*-tests and Fisher’s exact test (unadjusted results in Table 1). Between-group differences in primary and secondary study outcomes were assessed using analysis of covariance (ANCOVA), adjusting for covariates (baseline, age, and race/ethnicity; adjusted results in Table 2 and Table 3). Exercise identity (EI), ERI, HEI, HERI, and health behavior variables (i.e., intake of fruits and vegetables, and weekly participation in aerobic and resistance training activities) were analyzed to determine if the camp cohorts changed differently at post-camp and follow-up. The data residuals of each ANCOVA test were reviewed to determine acceptable normality. If the ANCOVA test data residuals did not meet acceptable normality, the original data were transformed utilizing Tukey’s ladder of power methodology (square-root transformations) [36]. Review of the data residuals histogram, scatter plots of residuals plotted against fitted (predicted), and Levene’s test were used to determine approximate equal standard deviations of each output variable while adjusting for the categorical variables (camp, race/ethnicity). All means, and standard deviations of transformed data have been reported in the untransformed format to enhance clarity for the reader (i.e., behavioral data are often reported in metrics such as “days per week” or “servings per day” which would be undistinguishable if transformed). The ANCOVA analyses of square-root transformed data are noted in Table 3.

## 3. Results

Participant demographics were well-matched between camps, however, a slight age difference across campers existed and the health promotion camp reported greater diversity. As expected, a significant difference in raw BMI values, as well as BMI *z*-scores and percentiles were observed between camps. Exercise role-identity (ERI) and healthy-eater role-identity (HERI) significantly differed at baseline. Table 1 provides comparisons of participant baseline characteristics per camp (unadjusted for covariates; see Table 2 and Table 3 for adjusted values).

Several significant differences in demographic characteristics were found between the health promotion cohort (*n* = 21) and obesity treatment cohort (*n* = 20) at baseline (denoted as mean[SD]), such as age (11.3 (0.97) vs. 12.40 (1.5) years respectively; *p* = 0.009), BMI *z*-score (0.136 (1.72) vs. 2.12 (0.490) respectively; *p* < 0.001), and race/ethnicity (greater diversity in health promotion cohort; *p* = 0.039). As expected, participants from the health promotion camp had significantly lower BMI than those from the obesity treatment camp. The difference in weight status across cohorts was an important component of the study design in determining potential weight-dependent disparities in health identity and behavior, and therefore no adjustment for difference in BMI was made in subsequent analyses. However, further analyses were adjusted for baseline, age and race/ethnicity (Table 2 and Table 3). The primary outcomes meaningfully differed at baseline, in agreement with the study hypothesis. Exercise identity (EI) and HEI was less salient in the obesity treatment cohort as compared to the health promotion cohort. Prior to the intervention, between-group differences in exercise role-identity (ERI) trended towards significant (*p* = 0.079) and statistically significant differences between camps were observed in healthy eating role-identity (HERI; *p* = 0.028) (adjusted for covariates; Table 2). As previously noted, role-identity in particular is strongly correlated to frequency of health behavior [31]. At baseline, the camp cohorts reported that 47.6% (health promotion) and 35.0% (obesity treatment) of the samples participated in at least 60 min of physical activity on 5+ days of the past seven days. Conversely, 14.3% (health promotion) and 20.0% (obesity treatment) reported exercising to strengthen or tone muscles on 3+ days of the past seven days at baseline. Frequency of fruit and vegetable intake per day differed across cohorts. At baseline, 61.9% and 47.6% of the health promotion sample reported eating 2+ daily servings of fruits and vegetables, respectively, as compared to only 45% (2+ servings of fruits per day) and 30% (2+ servings of vegetables per day) of the obesity treatment sample.

**Figure 2:**
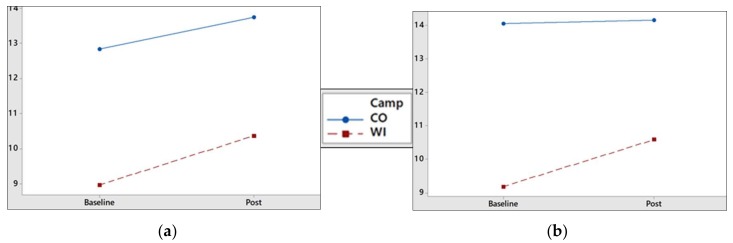
Visualization of differing changes in exercise and healthy eater role-identity between the Colorado (CO; health promotion) and Wisconsin (WI; obesity treatment) camps: (**a**) The magnitude of change from baseline to post-camp (Post) in ERI in the obesity treatment cohort appears greater than in the health promotion cohort; (**b**) An increase in HERI was observed in the obesity treatment cohort versus a plateau in HERI in the health promotion cohort (Baseline to Post).

Immediately following participation in the one-week behavioral health camp, EI, ERI, HEI, and HERI scores increased in both the health promotion and treatment cohorts. Changes in ERI, and perhaps most notably in HERI, in both cohorts at post-camp data collection is depicted in Figure 2. Changes in physical activity participation from baseline to post-camp were substantial, with 71.4% of the health promotion cohort and 85% of the treatment cohort reporting at least 60 min of exercise on 5+ days of the past seven days. Participation in resistance training also increased, with 76.2% of adolescent girls in the health promotion group reporting engaging in strengthening and toning exercises on 3+ days of the past seven days, and 35% of the obesity treatment group. Reported frequency of fruit and vegetable consumption remained consistent in the health promotion cohort (61.9% and 42.9% of the cohort reported consuming 2+ daily servings of fruits and vegetables, respectively), and increased to 75% reporting 2+ daily servings of fruits and vegetables, alike, in the obesity treatment cohort.

**Figure 3:**
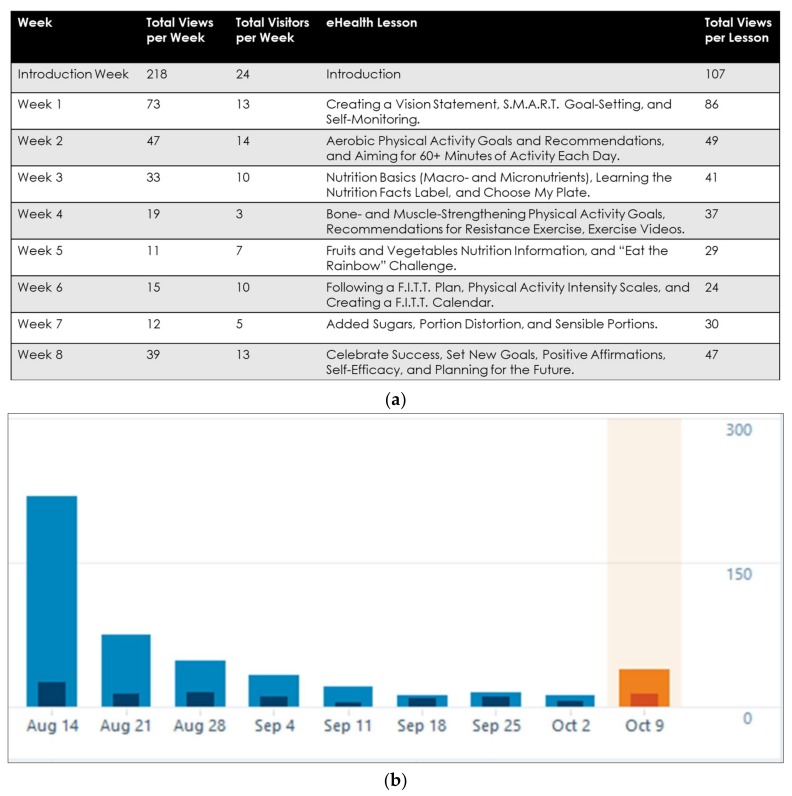
Overview of the eHealth program weekly lessons and webpage traffic: (**a**) total weekly views and visitors to the eHealth program peaked within the first few weeks; S.M.A.R.T. Goal-Setting denotes specific, measurable, attainable, realistic, and time-bound goals; F.I.T.T. Plan denotes frequency, intensity, type, and time chosen by the camper for physical activity participation (**b**) viewership and participation in the eHealth program peaked in the introductory week and dropped to a plateau throughout the eight-week curriculum; transparent bars represent total weekly views and opaque bars represent total weekly webpage visitors (the orange bar represents the final week of the eHealth program).

Participation in the optional eHealth program was low-to-moderate, as exemplified by online webpage traffic. No trends indicating significant differences between the health promotion and obesity treatment cohorts’ use of the eHealth were observed. The introductory period received the greatest magnitude of online traffic (107 views; 24 visitors), followed by the lessons delivered in Week 1 (86 views; 13 visitors), Week 2 (49 views, 14 visitors), and Week 8 (47 views; 13 visitors) (Figure 2). Overall, viewership of, and participation in, the eHealth program started high and dropped to a plateau throughout the duration of the eight-week curriculum (Figure 3). Completion rates of the adult caregiver feedback surveys were low, as were participant responses to the online health challenges. Email requests for final data collection resulted in a small follow-up subsample, of ten participants each, from the health promotion and obesity treatment cohorts. A two-sample *t*-test was performed comparing the baseline metrics of participants who completed both baseline and follow-up data, and those who did not submit follow-up data. Within each cohort, the participants who did not complete the entire study did not significantly differ from those who submitted follow-up data (*p* > 0.05). Therefore, the authors concluded that the follow-up subsample from each camp were representative of their cohort population.

Three-month follow-up health identity measurements revealed intriguing role-identity results. Overall, health identity appeared to marginally increase following the one-week camp experience in both the health promotion and obesity treatment cohorts and stabilize or even slightly decrease at follow-up. However, lasting shifts in role-identities (the sub-components of health identity which strongly predict future health behavior) were especially apparent in the obesity treatment cohort at follow-up. Exercise role-identity (ERI) and HERI scores in the obesity treatment cohort were substantially lower than the health promotion cohort at baseline. At follow-up, however, ERI and HERI in the obesity treatment cohort meaningfully increased from baseline, and role-identity scores no longer significantly differed between groups (Table 2).

Self-report participation in mild, moderate, and strenuous physical activity remained relatively consistent from baseline to follow-up in both the prevention and treatment cohorts, as did reported number of days per week the participants were active for at least 60 min. At follow-up, the health promotion cohort reported number of days per week that the participant did exercises to strengthen or tone muscles (i.e., push-ups, sit-ups, or weight lifting) as comparable to baseline (approximately 1–2 days per week at each data collection time-frame). However, the obesity treatment cohort showed substantially reduced self-report number of days engaged in strengthening/toning exercise from both baseline and post-camp levels (baseline and post-camp = approximately three days per week, reduced to less than one day per week at follow-up). Self-report intake of fruits and vegetables remained fairly stable from baseline to follow-up in both cohorts, although an initial significant difference in vegetable consumption between the health promotion and obesity treatment camps at baseline was attenuated at post-camp and follow-up. At follow-up, the obesity treatment cohort reported a small reduction in sugar-sweetened beverage (SSB) consumption. Several behavioral group differences were apparent immediately post-camp. Post-camp differences included the obesity treatment camp reporting significantly higher participation in 60+ min of physical activity per day, and significantly less engagement in exercises which strengthen or tone muscle (both variables were reported in days per week). Additionally, the obesity treatment group reported greater intake of fruits and vegetables (as measured by servings per day) immediately following participation in camp. Notably, the obesity treatment group reported practically zero consumption of SSB at post-camp (perhaps due to the dietary guidelines provided by the weight management camp). Table 3 depicts changes in physical activity and dietary variables, and group differences in self-report behavior between weight status cohorts. As previously noted, several variables have been transformed to meet normality (i.e., strenuous, moderate, and mild physical activity, and SSB), and the square-root transformed ANCOVA analyses are reported.

A secondary analysis examining potential correlations between select variables was conducted in both weight status cohorts at baseline and follow-up (Pearson’s coefficients and *p* values are reported in Table 4, Table 5, Table 6 and Table 7). Of particular interest were potential correlations between baseline BMI *z*-score, ERI, and HERI with secondary outcome variables (i.e., exercise and healthy eating behavior). In the health promotion cohort, BMI was not significantly correlated with health behavior at baseline but was moderately and negatively correlated with ERI (Table 4). In the obesity treatment group, BMI was also moderately and negatively correlated with ERI, as well as weight training and consumption of vegetables (Table 5). In both weight status cohorts, ERI and HERI were moderately and positively correlated to each other at baseline. A strong, negative relationship between baseline BMI *z*-score and ERI was apparent at follow-up in the health promotion group, as well as a moderate, negative relationship with HERI (Table 6). Also apparent within the health promotion group at follow-up was: (1) a strong, positive correlation between ERI and HERI, strenuous physical activity, and weight training; and (2) a moderate, positive correlation between HERI and the aforementioned exercise variables (Table 6). Within the obesity treatment cohort, a strong and positive relationship between (1) ERI and weight training, and (2) HERI and fruit and vegetable consumption was observed at follow-up (Table 7).

## 4. Discussion

The purpose of this study was to examine the effect of a brief, multicomponent behavioral intervention on differences in health identity between a sample of normal-weight and overweight or obese adolescent girls. Participation in a one-week camp experience, and optional eight-week eHealth program following camp, resulted in the mitigation or attenuation of role-identity score disparities between cohorts which were stratified according to weight status group at baseline. To the best of the authors’ knowledge, this the first study to investigate weight-dependent differences in the identity of ‘exerciser’ or ‘healthy eater’ (EI; HEI), as well as exercise and healthy eater role-identities (ERI; HERI: role-identity is particularly important as previous literature suggests this psychological variable is strongly correlated with frequency of, and motivation to participate in, healthy behaviors). Adolescent girls who do not regularly engage in physical activity or healthy eating practices may not possess a salient EI or HEI. A low EI and HEI may further predict inadequate levels of physical activity engagement and poor dietary practices as individuals age. Indeed, poorly developed health identities and subsequent avoidance or aversion to health behavior may habituate into adulthood and increase risk for a variety of health consequences associated with inactivity, poor dietary habits, and elevated BMI. Inversely, increasing EI and HEI in the teen years may cause adolescent girls to increase participation in habitual healthy behaviors, as they strive to remain in congruence with their embraced role-identity (i.e., “I am an exerciser, I am a healthy eater”). Ultimately, developing a strong EI and HEI may be an important strategy in both obesity prevention, and obesity treatment, programs for adolescent girls.

Results indicate that both the health promotion and obesity treatment cohorts experienced increases in EI and HEI following the respective one-week camps, with the obesity treatment group also experiencing a greater magnitude of change in ERI and HERI from baseline to post-camp. One potential explanation for the attenuation in ERI and HERI scores between the two groups at three-month follow-up is that the health promotion cohort reported more regular participation in physical activity and healthy dietary practices than the treatment sample, at baseline. Therefore, the health promotion cohort may have already possessed stable exercise and healthy eater role-identities at baseline measurement, and the brief intervention may not have significantly impacted already well-developed health identities [17,18]. This may be especially apparent when examining the plateau in HEI and HERI scores in the health promotion group at post-camp and follow-up (Figure 1 and Table 2). Conversely, the obesity treatment cohort may not have yet developed a stable ERI or HERI, and therefore closing the weight-dependent role-identity gap was plausible following a brief, multicomponent intervention.

Intriguing results from secondary analyses suggest that both ERI and HERI may be negatively correlated with BMI *z*-score, although the relationship strength appears most salient between ERI and BMI *z*-score. Notably, the strongest positive relationships observed in the health promotion cohort at follow-up were between ERI and exercise behaviors (i.e., strenuous physical activity and weight training). Within the obesity treatment cohort at follow-up, however, the most salient positive relationships were between HERI and healthy eating behaviors (i.e., fruit and vegetable consumption). Due to differences in the health programming emphasized at the two selected camps (health promotion camp = weight training and self-esteem vs. obesity treatment camp = behavior-change strategies for healthy weight management; Figure 1), it may be hypothesized that the campers who received a large volume of exercise training maintain a stronger relationship between ERI and exercise behavior than their peers who received extensive dietary guidance in regards to weight management (and vice versa). Further investigations into the specific mechanisms and interventional factors (i.e., nutrition-focused vs. physical-activity focused programing) that may underpin shifts in health identity are highly recommended by the authors.

Overall, the results from the current study are promising and consistent with previous literature indicating that behavioral health interventions may indeed enhance exercise and dietary behavior, and that EI and HEI may influence (or be influenced) by health behavior.

### Strengths and Limitations

Several limitations are present in our study, and therefore the results should be interpreted with caution. First, causality cannot be established due to the pilot study design, consisting of (*n* = 2) camps which were not randomized to stringently controlled intervention conditions. Several dissimilarities were apparent between the programming structures of each camp, as well as a range of differences between the camp participants. Thus, the differences in health identity changes between the two cohorts at follow-up may be linked to a variety of attributes. Moreover, identity and behavior are reciprocal in nature, thus concluding causation is problematic and should be contraindicated. Due to the population (i.e., a small pilot sample, all girls cohort) additional research is needed to determine generalizability of this intervention to other settings or populations (such as mixed-gender, all-male, diverse socioeconomic background or specific race/ethnicity cohorts). Furthermore, objective physical activity data should be collected to reduce the potential for self-report bias [37]. Further research is warranted to enhance participation in supplementary eHealth programs and increase study retention rates. Extending follow-up data collection to perhaps 2–3 years after the camp intervention may better elucidate the relationship between identity development in adolescence, long-term behavior-change, and health outcomes. Finally, the present study did not have a true control group and was selected utilizing convenience sampling. Randomization at the individual level may not be feasible within camp settings, and therefore cluster randomized trial designs are recommended for future research endeavors. Findings from the current pilot study should be replicated in future studies to confirm veracity of the results.

However, the novel study design encompassing both a normal-weight and overweight or obese sample is a major strength of this study and allows future researchers to substantially expand upon our findings. Data from the present study suggests that significant weight-dependent disparities in health identity are evident. If developed or strengthened, previous literature indicates that health identity may meaningfully influence and guide behavior (such as long-term participation in physical activity and healthy eating). Furthermore, our study adds to the literature by providing evidence that overweight or obese adolescent girls may significantly increase exercise and healthy eater role-identities after just one-week participation in a behavioral summer health camp. The aforementioned pilot data is invaluable in guiding future research endeavors in this understudied field.

## 5. Conclusions

Additional studies exploring longitudinal outcomes related to the interaction between EI, HEI, ERI, and HERI and health behavior are needed in normal-weight, overweight, and obese populations. Our results underscore the need to focus on psychological underpinnings of behavior in intervention programming, especially in adolescent years when identity is most saliently developed. The results from this study provide evidence that brief, multicomponent behavioral interventions may indeed impact health identity outcomes, and may mitigate weight-dependent disparities in exercise and healthy eater role-identity. The development of role-identities conducive to long-term exercise and healthy eating in teen years may create a foundation for life-long participation in healthy behavior and may be an important strategy to prevent and treat overweight and obesity. Further investigations are recommended.

## Figures and Tables

**Table 1:** Characteristics of adolescent girls at baseline, health promotion cohort (Colorado, CO camp), and obesity treatment cohort (Wisconsin, WI camp).

Characteristic	Health Promotion Cohort (CO) *n* = 21	Obesity Treatment Cohort (WI) *n* = 20	*p* Value	
Age, years (SD)	11.3 (0.97)	12.40 (1.47)	**0.009 ^t^**	**
Sex, female (%)	21 (100.0)	20 (100.0)	-	-
BMI, kg/m^2^ (SD)	20.19 (6.42)	31.57 (6.62)	**<0.001 ^t^**	***
BMI, *z*-score (SD)	0.136 (1.72)	2.12 (0.490)	**<0.001 ^t^**	***
BMI, percentile (SD)	57 (36)	97 (3)	**<0.001 ^t^**	***
Race/ethnicity (%)	Asian: 1 (4.8) Black/African-American: 1 (4.8) Hispanic/Latino: 4 (19.0) Native American: 1 (4.8) White: 14 (66.7)	Black/African-American: 1 (5.0) White: 19 (95.0)	**0.039 ^f^**	*
Exercise role-identity: ERI (SD)	12.86 (4.86)	8.85 (4.17)	**0.007 ^t^**	**
Healthy eater role-identity: HERI (SD)	13.76 (3.22)	9.25 (4.99)	**0.001 ^t^**	**

Results presented as mean (SD; standard deviation) or number (%); bold text denotes significance; BMI = body mass index; ERI = subset of EI scale (1,2,6); HERI = subset of HEI scale (1,2,6). * *p* < 0.05. ** *p* < 0.01 *** *p* < 0.001. t = two-sample *t*-test. f = Fisher’s exact test.

**Table 2:** Health identity variables at baseline, post-camp, and follow-up. Data are listed by camp.

Health Identity Variables	Health Promotion Cohort *n* = 21(BL-PC); 10(FU)	Obesity Treatment Cohort *n* = 20(BL-PC); 10(FU)	Group Differences *p* Value	
Exercise identity (all 9 EIS items)	BL: 40.43 (11.06)	BL: 36.60 (8.54)	BL: 0.709 ^a^	-
	PC: 42.33 (10.04)	PC: 38.60 (11.56)	PC: 0.146 ^a^	-
	FU: 39.20 (15.43)	FU: 33.20 (9.93)	FU: 0.297 ^a^	-
Exercise role-identity (EIS 1,2 6)	BL: 12.86 (4.86)	BL: 8.85 (4.17)	*BL: 0.079* ^a^	-
	PC: 13.62 (3.83)	PC: 10.12 (4.14)	PC: 0.308 ^a^	-
	FU: 12.40 (5.78)	FU: 9.20 (3.82)	FU: 0.254 ^a^	-
Healthy eater identity (all 9 HEI items)	BL: 43.19 (8.85)	BL: 39.90 (9.90)	BL: 0.579 ^a^	-
	PC: 43.33 (9.13)	PC: 40.95 (9.64)	PC: 0.859 ^a^	-
	FU: 38.70 (14.28)	FU: 40.70 (12.05)	FU: 0.041 ^a^	-
Healthy eater role-identity (HEI 1, 2, 6)	BL: 13.76 (3.22)	BL: 9.25 (4.99)	**BL: 0.028 ^a^**	*
	PC: 13.91 (2.81)	PC: 10.65 (4.37)	PC: 0.821 ^a^	-
	FU: 14.10 (4.33)	FU: 12.60 (4.62)	FU: 0.256 ^a^	-

Results presented as mean (SD; standard deviation); bold text denotes significance and italicized text trends towards significance; BL = baseline; PC = post-camp; FU = follow-up. * *p* < 0.05. ** *p* < 0.01 *** *p* < 0.001. a = ANCOVA Test.

**Table 3:** Physical activity and dietary variables at baseline, post-camp, and follow-up.

Physical Activity (PA) And Dietary Variables	Health Promotion Cohort (CO)	Obesity Treatment Cohort (WI)	Group Differences *p* Value	
Strenuous PA in hours per week	BL: 1.94 (1.77)	BL: 2.14 (2.20)	BL: 0.560 ^a,s^	-
	FU: 1.54 (1.33)	FU: 2.47 (2.54)	FU: 0.438 ^a,s^	-
Moderate PA in hours per week	BL: 2.90 (2.83)	BL: 2.67 (2.39)	BL: 0.109 ^a,s^	-
	FU: 1.47 (1.43)	FU: 2.04 (2.38)	FU: 0.579 ^a,s^	-
Mild PA in hours per week	BL: 2.85 (3.29)	BL: 3.14 (2.66)	BL: 0.946 ^a,s^	-
	FU: 2.84 (2.91)	FU: 3.27 (2.38)	FU: 0.909 ^a,s^	-
Days per week participant was physically active for 60+ min/day.	BL: 4.00 (2.10)	BL: 3.75 (1.77)	BL: 0.908 ^a^	-
	PC: 4.71 (1.71)	PC: 6.70 (0.92)	**PC: 0.001** ^a^	**
	FU: 3.30 (2.16)	FU: 3.90 (1.91)	FU: 0.885 ^a^	-
Days per week participant did exercises to strengthen or tone muscles (i.e., push-ups, sit-ups, or weight lifting).	BL: 1.81 (2.11)	BL: 2.75 (2.55)	BL: 0.743 ^a^	-
	PC: 4.95 (0.74)	PC: 3.00 (2.88)	**PC: 0.024** ^a^	*
	FU: 1.50 (2.32)	FU: 0.70 (1.49)	FU: 0.195 ^a^	-
Servings of fruit participant ate per day.	BL: 1.97 (1.25)	BL: 1.59 (1.37)	BL: 0.369 ^a^	-
	PC: 1.84 (1.10)	PC: 2.69 (1.38)	*PC: 0.052* ^a^	-
	FU: 1.80 (1.14)	FU: 1.07 (0.68)	FU: 0.118 ^a^	-
Servings of vegetables participant ate per day.	BL: 1.73 (1.37)	BL: 1.18 (1.07)	**BL: 0.016** ^a^	*
	PC: 1.34 (1.20)	PC: 2.49 (1.32)	**PC: 0.036** ^a^	*
	FU: 1.66 (1.19)	FU: 1.56 (0.88)	FU: 0.523 ^a^	-
Servings of sugar-sweetened beverages participant ate per day.	BL: 0.66 (0.85)	BL: 0.75 (1.21)	BL: 0.617 ^a,s^	-
	PC: 0.50 (0.87)	PC: 0.01 (0.06)	**PC: < 0.001** ^a,s^	***
	FU: 0.66 (0.54)	FU: 0.37 (0.35)	*FU: 0.059* ^a,s^	-

Results presented as mean (SD; standard deviation); bold text denotes significance and italicized text trends towards significance; BL = baseline; PC = post-camp; FU = follow-up. * *p* < 0.05. ** *p* < 0.01 *** *p* < 0.001. a = ANCOVA Test. s = square-root transformation.

**Table 4:** Pearson coefficient and *p* value of select variables demonstrating baseline correlations between ERI and HERI (positive; strong), strenuous physical activity and HERI (positive; moderate), and weight training and HERI (positive; moderate) in the health promotion cohort.

Health Promotion Cohort (CO) Baseline	1	2	3	4	5	6	7
1. BMI *z*-score	-						
2. Exercise role-identity (ERI)	**−0.458** *p* = 0.055	-					

3. Healthy eater role-identity (HERI)	−0.408 *p* = 0.092	0.747 *** ***p*** **< 0.001**	-				
				
4. Strenuous physical activity (hours per week)	0.061 *p* = 0.807	0.556 ** ***p*** **= 0.008**	0.415 *p* = 0.060	-			
			
5. Weight training (days per week)	−0.365 *p* = 0.135	0.323 *p* = 0.152	0.433 * ***p*** **= 0.049**	0.284 *p* = 0.211	-		
		
6. Servings of fruits (per day)	0.271 *p* = 0.275	0.061 *p* = 0.790	-0.007 *p* = 0.974	0.361 *p* = 0.107	-0.124 *p* = 0.590	-	
	
7. Servings of vegetables (per day)	−0.110 *p* = 0.663	−0.049 *p* = 0.829	−0.028 *p* = 0.903	0.065 *p* = 0.776	−0.011 *p* = 0.961	0.476 * ***p*** **= 0.028**	-


Bold text denotes significance and italicized text trends towards significance. * *p* < 0.05. ** *p* < 0.01 *** *p* < 0.001.

**Table 5:** Pearson coefficient and *p* value of select variables demonstrating baseline correlations between BMI and ERI (negative; moderate), ERI and HERI (positive; moderate), weight training and BMI (negative; moderate), servings of vegetables per day and BMI (negative; moderate), and servings of vegetables per day and ERI (positive; moderate) in the obesity treatment cohort.

Obesity Treatment Cohort (WI) Baseline	1	2	3	4	5	6	7
1. BMI *z*-score	-						
2. Exercise role-identity (ERI)	−0.5294 * ***p* = 0.035**	-					
					
3. Healthy eater role-identity (HERI)	0.299 *p* = 0.261	0.664 ** ***p* = 0.001**	-				
				
4. Strenuous physical activity (hours per week)	−0.060 *p* = 0.826	0.324 *p* = 0.163	0.301 *p* = 0.198	-			
			
5. Weight training (days per week)	−0.591 * ***p* = 0.016**	0.209 *p* = 0.377	0.241 *p* = 0.307	0.164 *p* = 0.489	-		
		
6. Servings of fruits (per day)	−0.215 *p* = 0.423	0.117 *p* = 0.622	0.263 *p* = 0.263	0.261 *p* = 0.266	0.578 ** ***p* = 0.008**	-	
	
7. Servings of vegetables (per day)	−0.561 * ***p* = 0.024**	0.520 * ***p* = 0.019**	0.316 *p* = 0.174	0.270 *p* = 0.250	0.315 *p* = 0.177	0.466 * ***p* = 0.039**	-


Bold text denotes significance and italicized text trends towards significance. * *p* < 0.05, ** *p* < 0.01, *** *p* < 0.001.

**Table 6:** Pearson coefficient and *p* value of select variables demonstrating follow-up correlations between BMI and ERI (negative; strong), BMI and HERI (negative; moderate), ERI and HERI (positive; strong), BMI and strenuous physical activity (negative; moderate), strenuous physical activity and ERI (positive; strong), and HERI (positive; moderate) in the health promotion cohort

Health Promotion Cohort (CO) Follow-Up	1	2	3	4	5	6	7
1. BMI *z*-score	-						
2. Exercise role-identity (ERI)	−0.850 ** ***p* = 0.004**	-					
					
3. Healthy eater role-identity (HERI)	−0.667 * ***p* = 0.049**	0.842 ** ***p* = 0.002**	-				
				
4. Strenuous physical activity (hours per week)	−0.680 * ***p* = 0.044**	0.793 ** ***p* = 0.006**	0.678 * ***p* = 0.031**	-			
			
5. Weight training (days per week)	**−0.589** *p* = 0.095	0.795 ** ***p* = 0.006**	0.691 * ***p* = 0.027**	0.653 * ***p* = 0.041**	-		
		
6. Servings of fruits (per day)	−0.278 *p* = 0.469	0.471 *p* = 0.170	0.502 *p* = 0.140	0.091 *p* = 0.803	0.422 *p* = 0.225	-	
	
7. Servings of vegetables (per day)	−0.274 *p* = 0.476	0.478 *p* = 0.163	0.495 *p* = 0.146	0.283 *p* = 0.428	0.691 * ***p* = 0.027**	0.803 ** ***p* = 0.005**	-


Bold text denotes significance and italicized text trends towards significance. * *p* < 0.05, ** *p* < 0.01, *** *p* < 0.001.

**Table 7:** Pearson coefficient and *p* value of select variables demonstrating follow-up correlations between ERI and weight training (positive; strong), HERI and weight training (positive; moderate), HERI and fruit (positive; strong), and vegetable consumption (positive; strong) in the obesity treatment cohort

Obesity Treatment Cohort (WI) Follow-Up	1	2	3	4	5	6	7
1. BMI *z*-score	-						
2. Exercise role-identity (ERI)	**−0.719** *p* = 0.069	-					
					
3. Healthy eater role-identity (HERI)	−0.201 *p* = 0.666	0.508 *p* = 0.134	-				
				
4. Strenuous physical activity (hours per week)	−0.202 *p* = 0.663	0.025 *p* = 0.946	−0.370 *p* = 0.292	-			
			
5. Weight training (days per week)	−0.649 *p* = 0.115	0.751 * ***p* = 0.012**	0.672 * ***p* = 0.033**	0.031 *p =* 0.932	-		
		
6. Servings of fruits (per day)	−0.133 *p* = 0.776	**0.582** *p* = 0.077	0.878 *** ***p* < 0.001**	−0.3401 *p* = 0.336	0.706 * ***p* = 0.022**	-	
	
7. Servings of vegetables (per day)	−0.241 *p* = 0.603	0.451 *p* = 0.191	0.754 * ***p* = 0.012**	−0.289 *p* = 0.418	**0.602** *p* = 0.065	**0.614** *p* = 0.059	-


Bold text denotes significance and italicized text trends towards significance. * *p* < 0.05, ** *p* < 0.01, *** *p* < 0.001.
